# Maternal mental disorders, psychotropic drugs, socioeconomic status, and offspring congenital anomalies

**DOI:** 10.1177/03000605241306902

**Published:** 2025-01-16

**Authors:** Satu-Maarit Björkstedt, Hannu Koponen, Hannu Kautiainen, Mika Gissler M, P. Pennanen, Johan G Eriksson, Merja K Laine

**Affiliations:** 1 Department of General Practice and Primary Health Care, University of Helsinki and Helsinki University Hospital, Helsinki, Finland; 2 Social Services and Health Care Division, City of Helsinki, Helsinki, Finland; 3 Department of Psychiatry, University of Helsinki and Helsinki University Hospital, Helsinki, Finland; 4 Folkhälsan Research Center, Helsinki, Finland; 5 Primary Health Care Unit, 60650 Kuopio University Hospital, Kuopio, Finland; 6 Finnish Institute for Health and Welfare, Helsinki, Finland; 7 Karolinska Institute and Region Stockholm, Stockholm, Sweden; 8 Wellbeing services county of Vantaa and Kerava, City of Vantaa and City of Kerava, Finland; 9 Department of Obstetrics and Gynecology and Human Potential Translational Research Program, Yong Loo Lin School of Medicine, National University Singapore, Singapore; 10 Agency for Science, Technology and Research (A*STAR), Singapore Institute for Clinical Sciences (SICS), Singapore, Singapore

**Keywords:** Congenital anomaly, mental disorder, pregnancy, primiparous, psychotropic drug, register-based cohort study, socioeconomic status

## Abstract

**Objective:**

To evaluate whether there is an association between maternal mental health, purchase of psychotropic drugs, socioeconomic status and major congenital anomalies in offspring.

**Methods:**

A register-based cohort study of 6189 Finnish primiparous women who had a singleton delivery between 2009 and 2015. Data on pregnancy and delivery outcomes, psychiatric diagnosis, prescription drug purchases and offspring congenital anomalies were obtained from Finnish national registers.

**Results:**

Severe depressive disorders were diagnosed in 2.0% of women and severe anxiety disorders in 1.1%. During pregnancy, 9.6% of women purchased psychotropic drugs. Of these women, 5.7% delivered an offspring with a major congenital anomaly. Women who purchased psychotropic drugs in pregnancy had an increased risk of delivering a child with major congenital anomalies compared with women who did not purchase psychotropic drugs. Multivariate regression analysis showed that purchase of benzodiazepines increased the risk of major congenital anomalies (odds ratio 2.11 [95% confidence interval 1.17 to 3.81]). Pregnant women purchasing psychotropic drugs more often lived alone and smoked, had higher body mass index, and had lower annual income and educational attainment than women not purchasing psychotropic drugs.

**Conclusions:**

Benzodiazepine use, but not socioeconomic status, may be associated with major congenital abnormalities in offspring.

## Introduction

Major congenital anomalies are not only associated with distress, but also lead to lifelong disabilities, increased mortality risks and societal costs.^1^ However, in two out of three cases, the etiology of such anomalies remains unknown.^1,2^ Various causes of congenital anomalies have been identified, including genetic defects, multifactorial inheritance, environmental teratogens and micronutrient deficiency.^1^ Major congenital anomalies are diagnosed in 2% to 5% of offspring globally, 5% of offspring in Finland, 3% to 5% in the USA and 2.5% in Europe.^3,4^ In Europe, the most prevalent types of major congenital anomalies are cardiac, kidney and urinary tract, and limb congenital anomalies.^5^

The prevalence of common mental disorders such as depression and anxiety in pregnant women varies from 16% to 45%.^6,7^ Of antidepressants, selective serotonin reuptake inhibitors (SSRI) are the most commonly used in pregnant women, with an estimated prevalence of 3% worldwide, 6% in North America and 2% in Europe.^8^ Furthermore, 2% to 4% of pregnant women purchase benzodiazepines (BZD)^9–11^ and 1% to 2% purchase antipsychotics.^12^ Of pregnant women who use antipsychotics, 62% purchase quetiapine.^13^ Data on the safety of psychotropic drugs in pregnancy are inconsistent and depend on several factors, including the specific drug studied, the timing of drug use, the characteristics and limitations of the study population, and the length of the follow-up period during which congenital anomalies are diagnosed.^14^ Fetal exposure to SSRI in pregnancy seems to be associated with no or only slightly increased risk for congenital anomalies,^15,16^ and non-SSRI antidepressants or BZD either slightly increase the risk or have no effect.^17^ Further, atypical findings from several large studies show that, as a class, second-generation antipsychotics are not associated with congenital malformations.^11,18,19^ Maternal low socioeconomic status (SES) seems to have a moderate association with an increased risk of congenital anomalies.^20–24^ However, study results are conflicting.^22^

Data are generally lacking on the association between the use of psychotropic drugs in pregnancy and SES and congenital anomalies. Our aim was to evaluate whether severe maternal mental disorders, purchases of psychotropic drugs (SSRI, BZD, quetiapine) in pregnancy, and SES were associated with congenital anomalies in offspring of primiparous women living in the fourth largest city in Finland.

## Material and methods

### Study design and participants

This was a cohort study conducted in the city of Vantaa, Finland, which is located in the Helsinki metropolitan area. In 2015, approximately 220,000 inhabitants lived in Vantaa. Of these, 20% were women of childbearing age. In this study, we included all Finnish primiparous women (N = 6189) with no previous diagnosis of diabetes mellitus who delivered a singleton between 1 January 2009 and 31 December 2015. Women were defined as Finnish if their native language was Finnish or Swedish. Women with pregestational diagnosis of diabetes mellitus were excluded to avoid the possibility of an independent association between diabetes and congenital anomalies.

### Ethical issues

The study was approved by the ethics committee of the Hospital District of Helsinki and Uusimaa, Finland (356/13/03/03/2015) and the health authority of the City of Vantaa, Finland. The Finnish Institute for Health and Welfare, the Finnish Social Insurance Institution, Statistics Finland, and the Finnish Tax Administration gave permission to use the register data in the study. Informed consent was not required and none of the study participants were contacted because this was a register-based cohort study.

### Data on women

The Finnish Medical Birth Register includes data on all births and stillbirths from 500 g or 22 gestational weeks onwards, pregnancy history (number of induced abortions, miscarriages, ectopic pregnancies), mother’s age at delivery, smoking and cohabitation status, pre-pregnancy weight and height, diagnoses during pregnancy and at birth, such as gestational diabetes mellitus, and fertility treatments.

Women who smoked or stopped smoking during pregnancy were considered to be smokers. Body mass index (BMI) prior to conception was calculated as the square of the pre-pregnancy weight (kg) and height (m). A 30-kg/m^2^ criterion was used to identify obesity. The date of conception was estimated based on the delivery date and length of the pregnancy. If a woman gave birth to a live child or a stillbirth for the first time, she was considered primiparous. A delivery that occurred before 37 + 0 gestational weeks was considered preterm. Cesarean sections were categorized as elective, urgent, or emergency.

We sourced information on maternal educational attainment from Statistics Finland. Four categories were used to categorize educational attainment: I basic education (9 to 10 years of education), II upper secondary 6 education/postsecondary non-tertiary education (11 to 14 years of education), III bachelor's degree education (15 to 16 years of education) and IV master's/doctoral education (≥17 years of education).

Annual data on taxable income, including both earned and capital income at the individual level, was provided by the Finnish Tax Administration. Preconception yearly income was defined as the mean taxable income for the year of consumption and the two preceding years. A 2020 consumer price index adjustment was made to the annual income (Statistics Finland; http://www.stat.fi/til/index_en.html).

Data on specialized outpatient visits and hospitalization care 1 year prior to conception were obtained from the Care Register for Health Care, which is maintained by the Finnish Institute of Health and Welfare, and coded according to the World Health Organization’s International Statistical Classification of Diseases and Related Health Problems 10th Revision (ICD-10) codes (https://icd.who.int/en). If a woman had one or more outpatient appointments or hospitalizations because of mental health disorders with ICD-10 codes F10–F61 and F90, her mental health condition was considered severe. The onset of severe mental disorders was defined as the day the psychiatric diagnosis was coded. In Finland, persons with non-severe mental disorders are treated primarily in primary health care.

We obtained data on chronic illnesses 3 years before conception from the Finnish Social Insurance Institution (http://www.kela.fi/web/en/reimbursements-for-medicine-expenses), which collects data on all reimbursed drug purchases. Finnish Social Insurance Institution Reimbursement claims require the treating physician to generate a medical certificate with the patient’s chronic disease history and observations of their current health status. After reviewing the medical certificate, the Social Insurance Institution physicians grant the patient the right to reimbursable medication if the chronic disease criteria are satisfied. Concurrently, the entitlement is entered into a national registry.

The Social Insurance Institution maintains a register of all prescription drug purchases with data on Anatomical Therapeutic Chemical classification (ATC) codes and purchase date. From this source, we obtained data on drug purchases with ATC codes and purchase dates. Women were defined as purchasing psychotropic drugs in pregnancy if their prescription drug purchases had ATC codes N06A (antidepressants), N05BA (BZD derivatives, anxiolytics), N05A (antipsychotics) and N05AH04 (quetiapine).

### Data on offspring

We obtained the following data on offspring characteristics from the Finnish Medical Birth Register: the child’s sex, birth weight and height, head circumference, and any hospitalizations for resuscitation in critical care, intubation, antibiotic therapy, or respiratory care before the age of 7 days. In this cohort, offspring birth weight was calculated as Z-scores based on gestational age and sex. The ponderal index was calculated by dividing the birth length (m^3^) by the birth weight (kg).

The Finnish Institute of Health and Welfare maintains the Register on Congenital Malformations, from which we obtained information on congenital anomalies diagnosed during the first year of life. We categorized the congenital anomalies using European guidelines (EUROCAT, https://eu-rd-platform.jrc.ec.europa.eu/eurocat/data-collection/guidelines-for-data-registration_en). Each anomaly was assessed by a medical doctor and coded using ICD-10 2016 Version codes for Congenital malformations, deformations, and chromosomal abnormalities (ICD-10 codes Q00–Q99). According to the EUROCAT guidelines, we excluded minor anomalies.

Finnish national medical registers are comprehensive, valid, and of high quality.^25^ The reporting of this study conforms to STROBE guidelines.^26^ All data were pseudonymized, and the individuals cannot be identified. The study was conducted in accordance with the Helsinki Declaration of 1975 as revised in 2013.

### Statistical analyses

The demographic and clinical data are presented as means with standard deviations and counts with percentages (%). Statistical comparisons were made using the t-test, Mann–Whitney test or chi-square test. Univariate and multivariate logistic regression models were used to study the determinants of major congenital anomalies. Penalized maximum likelihood logistic regression was used for situations in which the event of interest was rare. In the case of violation of the assumptions (e.g. non-normality) for continuous variables, a bootstrap-style approach or Monte Carlo p-values (small number of observations) for categorical variables were used. Missing values were handled using available-case analysis. All tests were two-tailed and the level of significance was set at p < 0.05. The Stata 18.0 statistical package (StataCorp LP, College Station, TX, USA) was used for the analysis.

## Results

Of the primiparous women, 229 (3.7%) delivered a child with at least one major congenital anomality. Table 1 shows the characteristics of the women with offspring without or with major congenital anomalies. Severe depressive disorders (ICD-10 codes F32–F39) were diagnosed in 2.0% of women and severe anxiety disorders (ICD-10 codes F40–F45) in 1.1%. In pregnancy, 9.6% of women purchased psychotropic drugs (ATC codes N06A, N05BA, N05A or N05AH04), 8.0% antidepressants (ATC code N06A), 3.2% BZD (ATC code N05BA) and 0.7% quetiapine (ATC code N05AH04). None of the women purchased valproate (ATC N03AG01). Table 2 shows the characteristics of the pregnant women according to whether or not they purchased psychotropic drugs. Women who purchased psychotropic drugs were more often cohabiting, were smokers, had lower educational attainment and income, and had higher BMI than women who did not purchase psychotropic drugs.

**Table 1.** Characteristics of primiparous women (N = 6189) with offspring without or with major congenital anomalies.

	Women with offspring without major congenital anomaliesn = 5960	Women with offspring with major congenital anomaliesn = 229	p-value
Age (years), mean (SD)	28.5 (5.2)	28.2 (5.0)	0.40^a^
Cohabiting, n (%)	4743 (80)	181 (79)	0.84^b^
Smoked in pregnancy, n (%)	1088 (18)	54 (24)	0.041^b^
Years of education, mean (SD)	13.5 (2.6)	13.2 (2.6)	0.078^c^
Yearly income, median (IQR) EUR	24,240 (15,488, 31,674)	24,038 (15,934, 31,776)	0.85^d^
Height (cm), mean (SD)	166 (6)	165 (6)	0.25^a^
Weight (kg), mean (SD)	66 (14)	66 (14)	0.95^a^
Pre-pregnancy body mass index (kg/m^2^), mean (SD)	24.1 (4.6)	24.2 (4.5)	0.72^a^
Obesity (body mass index ≥30.0 kg/m^2^), n (%)	647 (11)	33 (14)	
Gestational diabetes mellitus, n (%)	957 (16)	46 (20)	0.10^b^
Number of abortions, miscarriages, ectopic pregnancies, n (%)			0.11^e^
None	4786 (80)	174 (76)	
1	828 (14)	38 (17)	
2	257 (4)	15 (7)	
3	89 (1)	<5 (<2)	
Fertility treatment, n (%)	529 (9)	14 (6)	0.14^b^
Duration of pregnancy (weeks), mean (SD)	39.9 (1.9)	39.3 (2.6)	<0.001^a^
Cesarean section, n (%)	1294 (22)	64 (28)	0.025^b^
Morbidity,* n (%)			
Thyroid disease	37 (1)	<5 (<2)	0.18^e^
Rheumatic disease	69 (1)	7 (3)	0.010^b^
Pulmonary disease	186 (3)	5 (2)	0.42^b^
Inflammatory bowel disease	55 (1)	<5 (<2)	0.99^e^
Cardiovascular disease	<5 (0)	<5 (<2)	0.99^e^
Epilepsy	45 (1)	<5 (<2)	0.69^e^
Psychiatric medication by ATC code**, n (%)			
N06A	468 (8)	28 (12)	0.017^b^
N05BA	183 (3)	16 (7)	<0.001^b^
N05A	53 (1)	<5 (<2)	0.99^e^
N05AH04	40 (1)	<5 (<2)	0.67^e^
N06A or N05BA or N05AH04	561 (9)	34 (15)	0.006^b^
Mental, behavioral, or neurodevelopmental disorders***, n (%)			
F10–19	16 (0)	<5 (<2)	0.97^d^
F20–29	16 (0)	<5 (<2)	0.97^d^
F30–31	22 (0)	<5 (<2)	0.95^d^
F32–39	108 (2)	14 (6)	<0.001^b^
F40–45	64 (1)	6 (3)	0.030^b^
F50	15 (0)	<5 (<2)	0.98^b^
F60–61	24 (0)	<5 (<2)	0.89^b^
F90	5 (0)	<5 (<2)	0.99^b^

Statistical comparisons were made using the ^a^t-test, ^b^chi-square test, ^c^bootstrap-type t-test, ^d^Mann–Whitney test and ^e^chi-square test with Monte Carlo p-value. IQR, interquartile range; SD, standard deviation.

*Data on morbidity obtained from the register of reimbursement rights for chronic diseases maintained by the Social Insurance Institution, Finland.

**Anatomical Therapeutic Chemical (ATC) codes N06A (antidepressants), N05BA (benzodiazepine derivatives, anxiolytics), N05A (antipsychotics), N05AH04 (quetiapine) in pregnancy.

***ICD-10 = International Statistical Classification of Diseases and Related Health Problems 10th Revision: F10–F19 mental and behavioral disorders due to psychoactive substance use, F20–F29 schizophrenia, schizotypal and delusional disorders, F30–F31 bipolar affective disorders, F32–F39 depressive disorders, F40–F45 anxiety disorders, F50 eating disorders, F60–F61 disorders of adult personality and behavior, F90 disturbance of activity and attention.

**Table 2.** Characteristics of primiparous women (N = 6189) who did and did not purchase psychotropic drugs.

	Women who did not purchase psychotropic drugs*n = 5594	Women who purchased psychotropic drugs*n = 595	p-value
Age (years), mean (SD)	29 (5)	28 (6)	0.55^a^
Cohabiting, n (%)	4496 (80)	428 (72)	<0.001^b^
Smoked in pregnancy, n (%)	964 (17)	178 (30)	<0.001^b^
Years of education, mean (SD)	13.5 (2.6)	12.7 (2.7)	<0.001^c^
Yearly income, median (IQR), EUR	24,576 (16,126, 31,947)	20,573 (9345, 28,470)	<0.001^d^
Height (cm), mean (SD)	166 (6)	165 (6)	0.075^a^
Weight (kg), mean (SD)	66.1 (13.5)	68.4 (15.0)	<0.001^a^
Pre-pregnancy body mass index (kg/m^2^), mean (SD)	24.0 (4.5)	25.0 (5.0)	<0.001^a^
Obesity (body mass index ≥30.0 kg/m^2^), n (%)	579 (10)	101 (17)	
Gestational diabetes mellitus, n (%)	881 (16)	122 (21)	0.003^b^
Number of abortions, miscarriages, ectopic pregnancies, n (%)			<0.001^b^
None	4514 (81)	446 (75)	
1	769 (14)	97 (16)	
2	232 (4)	40 (7)	
3	79 (1)	12 (2)	
Fertility treatment, n (%)	505 (9)	38 (6)	0.030^b^
Duration of pregnancy, (weeks), mean (SD)	39.9 (1.9)	39.8 (2.0)	0.34^a^
Cesarean section, n (%)	1199 (21)	159 (27)	0.003^b^
Morbidity, ** n (%)			
Thyroid disease	37 (1)	<5 (<1)	0.99^e^
Rheumatoid disease	71 (1)	5 (1)	0.37^b^
Pulmonary disease	170 (3)	21 (4)	0.51^b^
Inflammatory bowel disease	50 (1)	7 (1)	0.49^b^
Cardiovascular disease	<5 (0)	<5 (<1)	0.99^e^
Epilepsy	39 (1)	8 (1)	0.10^b^
Psychiatric disease	24 (0)	32 (5)	<0.001^b^
Mental, behavioral, or neurodevelopmental disorders, n (%)***	85 (2)	125 (21)	<0.001^b^
F10–F19	10 (0)	6 (1)	
F20–F29	<5 (0)	13 (2)	
F30–F31	6 (0)	16 (3)	
F32–F39	48 (1)	74 (12)	
F40–F45	9 (1)	41 (7)	
F50	6 (0)	9 (2)	
F60–F61	8 (0)	17 (3)	
F90	<5 (<1)	<5 (<1)	

Statistical comparisons were made using the ^a^t-test, ^b^chi-square test, ^c^bootstrap-type t-test, ^d^Mann–Whitney test and ^e^chi-square test with Monte Carlo p-value. IQR, interquartile range; SD, standard deviation.

* Prescription drug purchase with Anatomical Therapeutic Chemical (ATC) codes N06A (antidepressants), N05BA (benzodiazepine derivatives, anxiolytics), or N05AH04 (quetiapine) in pregnancy.

** Data on morbidity obtained from the register of reimbursement rights for chronic diseases maintained by the Social Insurance Institution, Finland.

*** ICD-10, International Statistical Classification of Diseases and Related Health Problems 10th Revision: F10–19 mental and behavioral disorders due to psychoactive substance use, F20–29 schizophrenia, schizotypal and delusional disorders, F30–31 bipolar affective disorders, F32–39 depressive disorders, F40–45 anxiety disorders, F50 eating disorders, F60–61 disorders of adult personality and behavior, F90 disturbance of activity and attention.

Of pregnant women who did purchase psychotropic drugs, 5.7% delivered a child with major congenital anomalies. For these women, the odds ratio (OR) of delivering a child with major congenital anomalies compared with women who did not purchase psychotropic drugs was 1.56 (95% confidence interval [CI] 1.06 to 2.29, adjusted for age, educational attainment, BMI, smoking, gestational diabetes mellitus, and pregnancy duration). Table 3 shows the pregnancy and perinatal outcomes according to offspring characteristics of women who purchased psychotropic drugs and those who did not. The offspring of women who purchased psychotropic drugs had increased risk of neonatal intensive care unit treatment before the age of 7 days (p < 0.001).

**Table 3.** Pregnancy and perinatal outcomes of offspring of primiparous women who did or did not purchase psychotropic drugs.

	Offspring of women who did not purchase psychotropic drugs*n = 5594	Offspring of women who purchased psychotropic drugs*n = 595	p-value
Boys, n (%)	2923 (52)	287 (48)	0.062^a^
Birth weight (g), mean (SD)			
Girls	3372 (546)	3333 (555)	0.24^b^
Boys	3473 (553)	3463 (622)	0.78^b^
Birth length (cm), mean (SD)			
Girls	49.5 (2.7)	49.1 (2.7)	0.042^b^
Boys	50.3 (2.5)	49.9 (3.0)	0.042^b^
Birth weight (Z-score), mean (SD)	0.00 (0.99)	−0.02 (1.03)	0.61^b^
Ponderal index (kg/m^3^), mean (SD)	27.4 (2.5)	27.7 (2.8)	0.004^b^
Head circumference (cm), mean (SD)			
Girls	34.6 (1.8)	34.4 (1.8)	0.17^b^
Boys	35.1 (1.8)	34.9 (2.1)	0.031^b^
Apgar score, 1 minute, mean (SD)	8.5 (1.4)	8.4 (1.5)	0.15^c^
Preterm birth <37 gestational weeks, n (%)	330 (6)	36 (6)	0.89^a^
Before the age of 7 days, n (%)			
Neonatal intensive care unit treatment	593 (11)	91 (15)	<0.001^a^
Respirator treatment	91 (2)	11 (2)	0.69^a^
Antibiotic treatment	362 (6)	51 (9)	0.051^a^
Preterm birth <37 gestational weeks, n (%)	330 (6)	36 (6)	0.89^a^

Statistical comparisons were made using the ^a^chi-square test, ^b^t-test and ^c^bootstrap-type t-test. SD, standard deviation.

* Prescription drug purchase with Anatomical Therapeutic Chemical (ATC) codes N06A (antidepressants), N05BA (benzodiazepine derivatives, anxiolytics), or N05AH04 (quetiapine) in pregnancy.

The major congenital anomalies, deformations, and chromosomal abnormalities according to ICD-10 codes of offspring born to women who did or did not purchase psychotropic drugs are shown in Table 4. Congenital anomalies of the circulatory and urinary systems were most frequently observed. A comparison of the group distributions could not be performed because of the small number of observations. Table 5 shows the covariates that explained major congenital anomalies. The multivariate analysis showed that purchase of BZD increased the risk of major congenital anomalies (OR 2.11 [95% CI 1.17 to 3.81]). The absolute difference between BZD purchasers and non-purchasers was 4.5% (95% CI 0.7 to 8.3).

**Table 4.** Major congenital anomalies, deformations, and chromosomal abnormalities by ICD-10 classification in offspring of primiparous women who did or did not purchase psychotropic drugs in pregnancy.

ICD-10 code	All offspringN = 6189	Offspring of women who did not purchase psychotropic drugs*n = 5594	Offspring of women who purchased psychotropic drugs*n = 595	OR (95 % CI)	p-value
Q00–Q07	7 (0.1)	6 (0)	<5 (<1)	2.17 (0.37 to 12.83)	0.51
Q10–Q18	17 (0.3)	15 (0)	<5 (<1)	1.52 (0.40 to 5.78)	0.68
Q20–Q28	124 (2.0)	109 (2)	15 (3)	1.34 (0.78 to 2.29)	0.34
Q30–Q34	5 (0.1)	<5 (0)	<5 (<1)	13.21 (2.60 to 67.12)	0.008
Q35–Q37	11 (0.2)	9 (0)	<5 (<1)	2.48 (0.61 to 10.00)	0.29
Q38–Q45	5 (0.1)	5 (0)	<5 (<1)	0.85 (0.05 to 15.45)	0.99
Q50–Q56	5 (0.1)	<5 (0)	<5 (<1)	13.21 (2.60 to 67.12)	0.008
Q60–Q64	11 (0.2)	8 (0)	<5 (<1)	3.88 (1.11 to 13.52)	0.081
Q65–Q79	60 (1.0)	49 (1)	11 (2)	2.20 (1.15 to 4.21)	0.021
Q80–Q89	12 (0.2)	11 (0)	<5 (<1)	1.22 (0.22 to 6.73)	0.99
Q90–Q99	5 (0.1)	5 (0)	<5 (<1)	0.85 (0.05 to 15.45)	0.98
Multiple	21 (0.3)	16 (0)	5 (1)	3.15 (1.20 to 8.30)	0.045

Statistical analysis using penalized maximum likelihood logistic regression. Unless otherwise stated, values are n (%). CI, confidence interval; ICD-10, International Statistical Classification of Diseases and Related Health Problems 10th Revision; OR, odds ratio.

*Anatomical Therapeutic Chemical (ATC) codes N06A (antidepressants), N05BA (benzodiazepine derivatives, anxiolytics), or quetiapine (N05AH04).

ICD-1- codes:

Q00–Q07 Congenital malformations of the nervous system

Q10–Q18 Congenital malformations of the eye, ear, face and neck

Q20–Q28 Congenital malformations of the circulatory system

Q30–Q34 Congenital malformations of the respiratory system

Q35–Q37 Cleft lip and cleft palate

Q38–Q45 Other congenital malformations of the digestive system

Q50–Q56 Congenital malformations of the genital organs

Q60–Q64 Congenital malformations of the urinary system

Q65–Q79 Congenital malformations and deformations of the musculoskeletal system

Q80–Q89 Other congenital malformations

Q90–Q99 Chromosomal abnormalities, not elsewhere classified

**Table 5.** Covariates explaining major congenital anomalies in offspring of primiparous women.

	UnivariateOR (95% CI)	MultivariateOR (95% CI)
Purchase of antidepressants	1.63 (1.09 to 2.45)	1.24 (0.78 to 1.98)
Purchase of benzodiazepine derivatives, anxiolytics	2.37 (1.40 to 4.02)	2.11 (1.17 to 3.81)
Purchase of quetiapine	1.61 (0.44 to 5.80)	0.79 (0.18 to 3.43)
Age	0.99 (0.96 to 1.01)	0.99 (0.96 to 1.02)
Education	0.95 (0.91 to 1.01)	0.98 (0.92 to 1.04)
Smoking	1.38 (1.01 to 1.89)	1.21 (0.85 to 1.72)
Body mass index	1.01 (0.98 to 1.03)	0.99 (0.97 to 1.02)
Duration of pregnancy	0.90 (0.85 to 0.94)	0.90 (0.85 to 0.94)
Gestational diabetes mellitus	1.31 (0.94 to 1.83)	1.28 (0.90 to 1.82)

CI, confidence interval; OR, odds ratio.

## Discussion

The purchase of BZD appeared to be associated with major congenital anomalies, whereas low SES was not. Pregnant women who purchased psychotropic drugs had several characteristics of low SES, such as living alone, smoking, elevated BMI, and low levels of educational attainment and income.

In our cohort, 3% of pregnant women were diagnosed with at least one severe mental disorder, most commonly depression and anxiety, as expected. This observation is in line with recent reports of the prevalence of anxiety disorders (3%), depression (3% to 5%), and psychotic disorders (2% to 4%) in pregnancy.^7^ We found that 10% of the pregnant primiparous women purchased psychotropic drugs. Previous studies have reported that 3% to 8% of pregnant women use antidepressants, 2% use atypical antipsychotics, and 2% to 4% use BZD.^8,9,18^

At the population level, major congenital anomalies have been observed in 2% to 5% of all pregnancies in recent decades.^3,4,27^ In our study cohort, the overall prevalence of major congenital anomalies was just below 4%. Of women who purchased psychotropic drugs, almost 6% delivered a child with major congenital anomalies compared with 3.5% of women who did not purchase psychotropic drugs. Thus, our findings support those of previous studies.^1–4^ Furthermore, we observed that women who purchased BZD had an increased risk of delivering a child with major congenital anomalies, but such an association was not observed with the other studied drugs. Several studies have focused on the association between use of BZD in the first trimester and congenital anomalies; however, the results are conflicting and absolute risks of BZD use in pregnancy are considered low.^14,28^ Use of alprazolam in early pregnancy may increase the risk of cardiovascular anomalies, and BZD as a class may cause pyloric stenosis or cleft lip and palate.^11,14^ Furthermore, BZD are often used in combination with other psychotropic drugs, which makes it challenging to evaluate the underlying reasons behind the possible congenital anomalies.^11,17^ Several pre-2005 studies on SSRIs found no association with major congenital anomalies, but more recent studies have shown an increased risk of cardiac anomalies in users of fluoxetine and paroxetine, and for neural tube and musculoskeletal anomalies in users of sertralin.^29^ Of the atypical antipsychotics, quetiapine is the most used drug in pregnancy,^12,19^ and appears to be a safe choice.^12–13^ Our study findings are partly in line with these results.

Previous study findings are controversial regarding the association between SES and risk of cardiovascular, orofacial cleft, musculoskeletal, facial and eye, and gastrointestinal anomalies, as well as for hernia and undescended testes.^21–24,30^ We found no association between the maternal characteristics of low SES and major congenital anomalies.

Our study had several strengths. The study cohort was comprehensive as it included all Finnish primiparous women who met the inclusion criteria from Finland’s fourth most populous city over a period of 7 years. The data on severe mental disorders can be considered reliable as they were based on diagnoses made by psychiatrists. Additionally, the information on prescription drug purchases was extensive and included prescriptions from hospitals, private clinics and primary health care providers.

There were some study limitations. In Finland, drug purchases reflect drug use; however, we lacked some information about the drugs, including dosage, duration of treatment and time point of use. Furthermore, it is important to acknowledge that some other diseases and drugs may be associated with congenital anomalies. We were also missing data on alcohol and substance use owing to poor reporting to the registers. Finally, because all study participants had a Finnish background, the findings have limited generalizability.

In conclusion, in primiparous women, purchases of BZD may be associated with major congenital anomalies in offspring, whereas the characteristics of low SES have no such association. Special consideration should be made when prescribing BZD to pregnant women.

## Data Availability

Sharing data is prohibited on ethical and legal grounds. The license applicant may only use data from the Finnish Institute for Health and Welfare, Statistics Finland, and the Social Insurance Institution for the purposes specified in the license agreement, which is social science research.
